# Mesenchymal stem cells limit vascular and epithelial damage and restore the impermeability of the urothelium in chronic radiation cystitis

**DOI:** 10.1186/s13287-022-03230-2

**Published:** 2023-01-11

**Authors:** Clément Brossard, Anne-Laure Pouliet, Anne‐Charlotte Lefranc, Mohamedamine Benadjaoud, Morgane Dos Santos, Christelle Demarquay, Valerie Buard, Marc Benderitter, Jean-Marc Simon, Fabien Milliat, Alain Chapel

**Affiliations:** 1 Institut de Radioprotection et de Sûreté Nucléaire (IRSN), PSE-SANTE/SERAMed/LRMed, 92260 Fontenay-aux-Roses, France; 2 Institut de Radioprotection et de Sûreté Nucléaire (IRSN), PSE-SANTE/SERAMed, 92260 Fontenay-aux-Roses, France; 3 Institut de Radioprotection et de Sûreté Nucléaire (IRSN), PSE-SANTE/SERAMed/LRAcc, 92260 Fontenay-aux-Roses, France; 4 Département de Radiothérapie Oncologie, APHP, Hôpital Universitaire Pitié-Salpêtrière, 47-83 Boulevard de l’Hôpital, 75651 Paris Cedex 13, France

**Keywords:** Chronic radiation cystitis, Mesenchymal stem cell, Cell therapy

## Abstract

**Background:**

Cellular therapy seems to be an innovative therapeutic alternative for which mesenchymal stem cells (MSCs) have been shown to be effective for interstitial and hemorrhagic cystitis. However, the action of MSCs on chronic radiation cystitis (CRC) remains to be demonstrated. The aim of this study was to set up a rat model of CRC and to evaluate the efficacy of MSCs and their mode of action.

**Methods:**

CRC was induced by single-dose localized irradiation of the whole bladder using two beams guided by tomography in female Sprague–Dawley rat. A dose range of 20–80 Gy with follow-up 3–12 months after irradiation was used to characterize the dose effect and the kinetics of radiation cystitis in rats. For the treatment, the dose of 40 Gy was retained, and in order to potentiate the effect of the MSCs, MSCs were isolated from adipose tissue. After expansion, they were injected intravenously during the pre-chronic phase. Three injections of 5 million MSCs were administered every fortnight. Follow-up was performed for 12 months after irradiation.

**Results:**

We observed that the intensity and frequency of hematuria are proportional to the irradiation dose, with a threshold at 40 Gy and the appearance of bleeding from 100 days post-irradiation. The MSCs reduced vascular damage as well as damage to the bladder epithelium.

**Conclusions:**

These results are in favor of MSCs acting to limit progression of the chronic phase of radiation cystitis. MSC treatment may afford real hope for all patients suffering from chronic radiation cystitis resistant to conventional treatments.

## Background

Interstitial, radiation and hemorrhagic cystitis have high incidence rates and treatments used for the most severe forms may be palliative. Previous studies have shown that mesenchymal stem cells (MSC) therapy is an alternative treatment for interstitial cystitis and hemorrhagic cystitis [1–4]. MSC treatment of radiation cystitis, on the other hand, has not yet been studied. However, the similar mechanisms of these pathologies favor a protective effect of MSCs on the chronic evolution of radiation cystitis [5]. Interstitial cystitis (IC) is an idiopathic disease which affects over one million people worldwide [6, 7]. Hemorrhagic cystitis (HC) is due to a wide variety of etiologies, including radiotherapy, with a frequency of 5–10% in the 6–20 years after radiation therapy [8] and with an incidence of between 7 and 68% [9–13] for hematopoietic stem cell transplantation. Chronic radiation cystitis (CRC) is a lesion of the bladder resulting from radiation therapy (prostate, colon or cervix).

CRC is characterized by vascular damage, epithelial injury, incontinence, hematuria, pelvic pain and, in the most severe cases, fistulas and death [14]. Vascular and urothelial damage contribute significantly to the progression of radiation cystitis in its chronic form. In CRC, as in other chronic radiation diseases, the progression of radiation-induced vascular and tissue damage is due in part to early events in endothelial cells. The slow regeneration of the endothelium could be the reason for the late vascular damage during the chronic phase [15]. These vascular lesions are characterized by telangiectasias and rupture of some blood vessels, creating microhemorrhages [8, 9].

In other types of cystitis, treatment with MSCs induces neovascularization, which could participate in the reduction of hematuria [2, 16–18]. The urothelium is the source of most cystitis in the acute phase. Its loss of impermeability leads to deep damage to the bladder wall. Imperfect repair of the urothelium leads to long-term damage, which is responsible for the chronic phases of cystitis [5]. Lesions of the urothelium are mainly due to decreased uroplakin expression and hyperplasia, both of which promote a loss of impermeability of the urothelium [19, 20]. Preclinical studies of MSC treatment of interstitial and hemorrhagic cystitis have shown that MSCs protect the urothelium from these injuries [1, 17, 21–23]. Furthermore, the action of MSCs on regeneration of irradiated epithelia has been demonstrated at the intestinal level [24].

Currently, no effective treatment is available and MSCs could be an alternative treatment. For CRC, a compassionate study of treatment for four over-irradiated patients from the Epinal radiotherapy accident supports a reduction in hematuria [2]. These results need to be confirmed by a preclinical study and the action of MSCs on vascular and urothelial damage needs to be characterized during the chronic evolution of radiation cystitis.

Based on the results of previous studies on cystitis and irradiated healthy tissue, we investigated the role of MSCs on vascular and urothelial injury in order to establish whether cell therapy is an effective therapeutic alternative for the treatment of CRC. We have developed a rat model of chronic radiation cystitis using single-dose bladder irradiation. Intravenous MSC treatment was evaluated for urothelial and vascular damage using physiological and tissue approaches.

## Methods

### Ethics approval and consent to participate

Animal experiments were performed in compliance with French and European regulations on protection of animals used for scientific purposes (EC Directive 2010/63/EU and French Decret 2013–118). Whole study was approved by a local ethics committee #81 “Comité d’Ethique en Expérimentation Animale” which belong to IRSN and authorized by the French Ministry of Research under the reference APAFIS#12922-201801051531419 v1, #17561-201801051531419 v2 (internal project number P17-09) and #20993-201906071027312 v2 (internal project number P19-09)]. Animals were housed in the IRSN animal facilities accredited by the French Ministry of Agriculture for performing experiments on live rodents.

### Animals

**Fig. 1:**
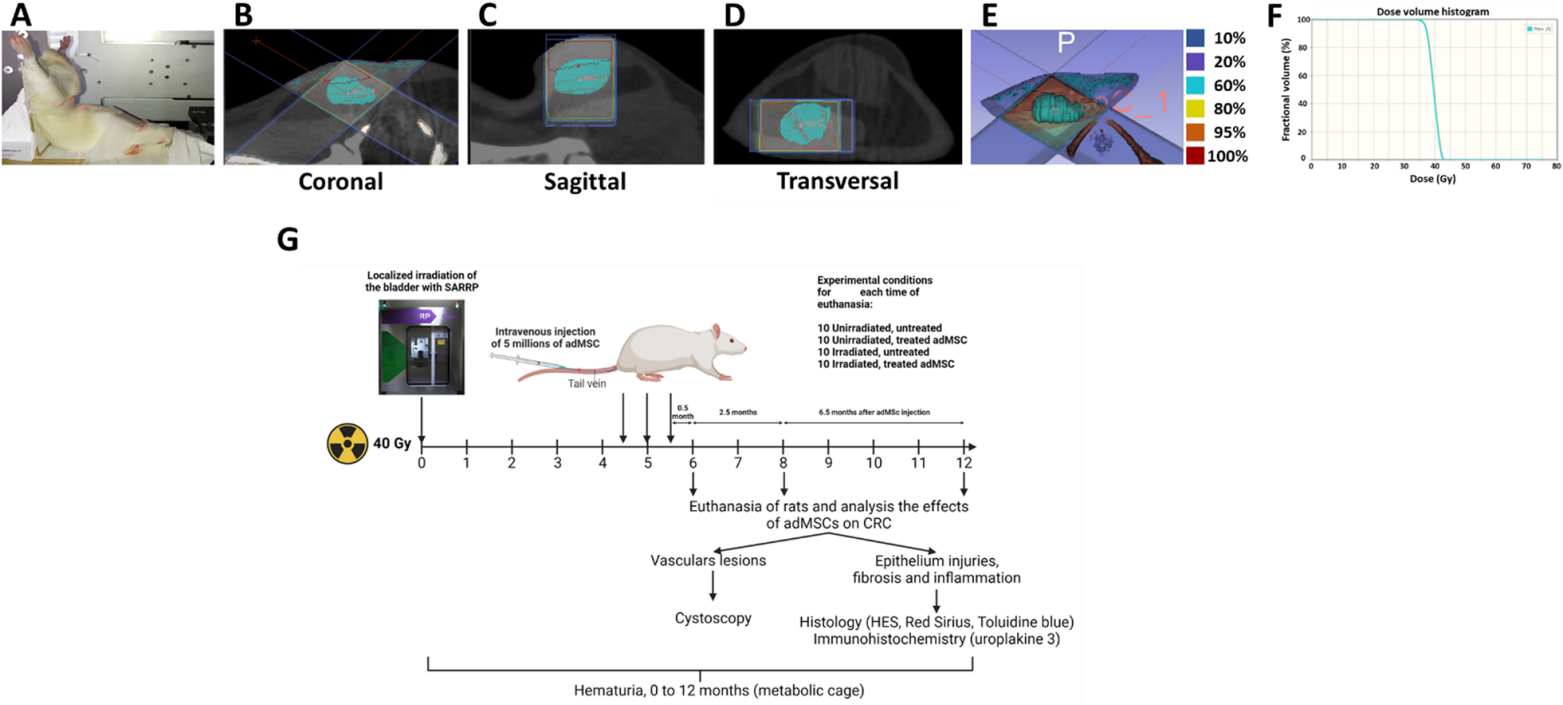
Irradiation configuration with the Small Animal Radiation Research Platform (SARRP) and study of hematuria. In the pictures, the bladder is represented in blue. **A** Placement of the animal in the SARRP. **B** Coronal view of the bladder. **C** Sagittal view of the bladder. **D** Transverse view of the bladder. **E** Representation of the isodose distribution in the tissue for a dose of 40 Gy. The bladder is represented in blue and receives 100% of the dose. **F** Histogram of the dose per volume in the bladder for a dose of 40 Gy. **G** Experimental design for studies with MSC treatment with a dose of 40 Gy and 12 months follow-up. Injections of 5 millions of MSC are performed at 4.5, 5 and 5.5 months post-irradiation. Euthanasia times are at 6, 8 and 12 months post-irradiation and tissue analyses are performed. Cystoscopies are performed at 6 and 12 months post-irradiation

To study the dose effect of bladder irradiation on the kinetics of radiation cystitis, 120 female rats were irradiated at doses from 20 to 80 Gy (20, 30, 40, 50, 60, 70, 80 Gy) and were followed up over the next 10 months. To evaluate the treatment using MSCs, the rats were then divided into three groups of 30 rats each. The animals were housed in double-decker cages, three or four to a cage, with access to food and water ad libitum and with light and dark cycles. Every effort was made to minimize suffering by adding enrichment such as wooden sticks and tunnels and all experiments were performed under gaseous anesthesia with isoflurane (Aerrane, Baxter SA, Lessines, Belgium). Animal behavioral and physiological parameters (e.g., bleeding and diarrhea) were monitored daily and suffering animals were euthanized. Euthanasia was performed by cardiac puncture under isoflurane anesthesia. Female Sprague–Dawley rats from Janvier Labs (Le Genest-Saint-Isle, France) were aged 10 weeks (250–300 g) at the beginning of the experiment. The protocol is described in further detail below and in Fig. 1. Rats that died before the planned euthanasia times were removed from the study. The experimental unit is a single animal. The control groups are the unirradiated untreated and unirradiated treated animals. At the reception of the animals, traceability and individual identification of animals were achieved using microchip implants. Animals were randomly distributed in the cages of the different groups. The experimenters were aware during the allocation, the conduct of the experiment, the outcome assessment, and the data analysis.

### Radiation schedule

Irradiation was performed using the SARRP (Small Animal Radiation Research Platform) from Xstrahl Life Sciences (XSTRAHL Ltd., Camberley, UK) with a voltage of 220 kV, an intensity of 13 mA, a dose rate of 2.4 Gy per minute, a collimator of 10 × 10 mm^2^ and a source-sample distance of 35 cm. An inherent filtration of 0.8 mm Beryllium and an additional filtration of 0.15 mm Copper (half-value layer = 0.667 mm Copper) were used. A CT scan was performed to visualize the position of the bladder and place the isocenter. Segmentations and dose calculations were performed with Muriplan software. Two irradiation beams were used with an angle between 90 and 120°. The animals were anesthetized and maintained with isoflurane (between 2 and 3.5% depending on the animal, with an air flow of 0.4 L/minute) and then placed in the SARRP. The rats were placed on their backs with their legs raised and spread, reminiscent of the supine Mézières position, and then the animals were held in position using adhesive plasters (see Fig. 1A–F).

To determine the optimal irradiation dose and to study the kinetics of radiation cystitis, 120 animals received single-dose irradiation of 0, 20, 30, 40, 50, 60, 70 and 80 Gy. For each irradiation dose, five animals were euthanized at 6 and 10 months post-irradiation.

**Fig. 2:**
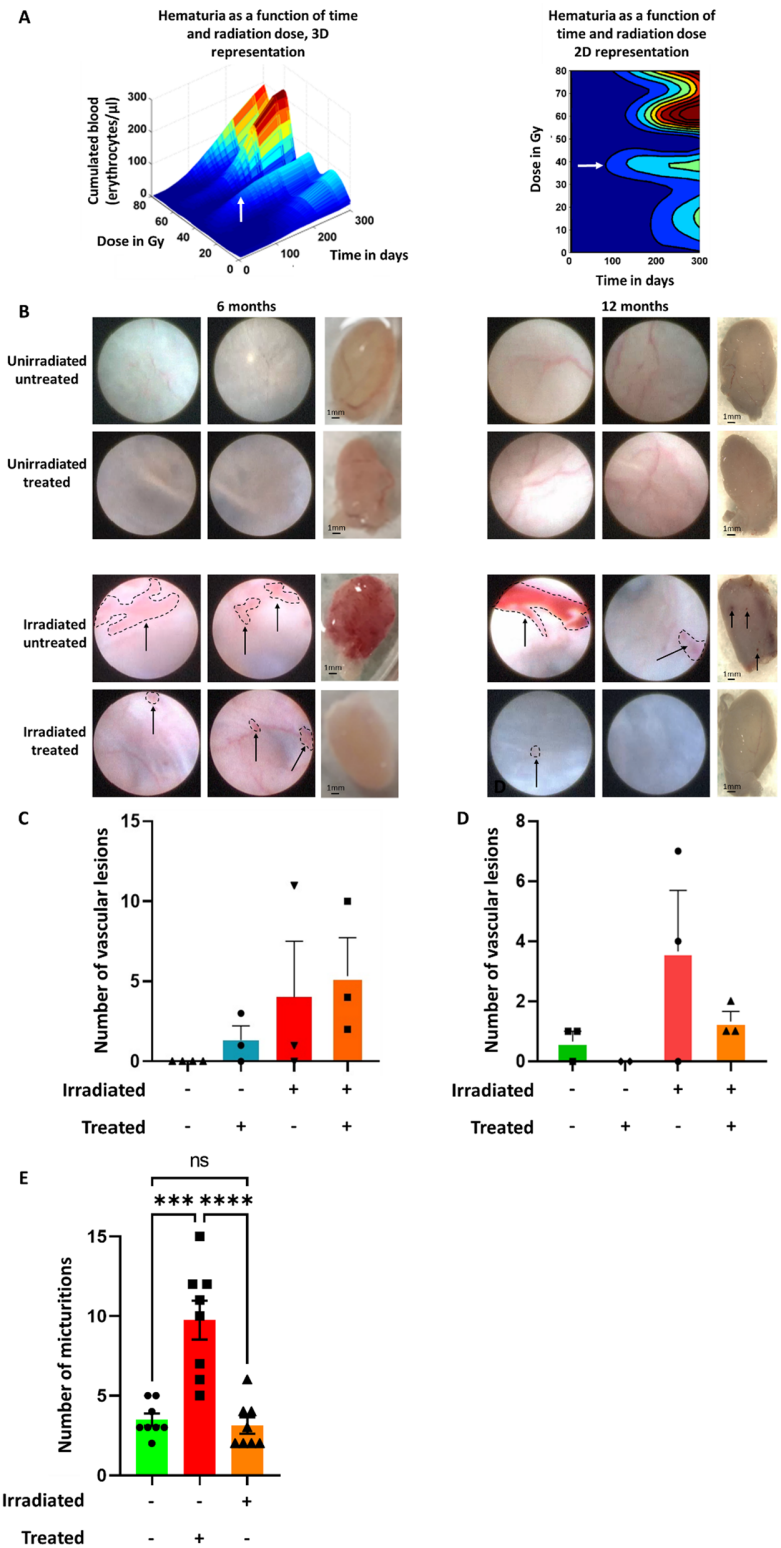
Analysis of hematuria, vascular lesions and micturition. **A** 3D and 2D representations of hematuria accumulation as a function of post-irradiation time and radiation dose. The white arrow indicates the dose threshold of hematuria onset within 100 days (for each group *n* = 5). **B** Cystoscopic studies of vascular lesions at 6 and 12 months post-irradiation. On the left are shown the rats 6 months post-irradiation and on the right the rats 12 months post-irradiation. The photographs shown on the left and in the center of each section are photographs taken during cystoscopy to visualize the vascular lesions. As they represent a three-dimensional image of the lesions, it is not possible to put scale bars. The images on the right represent the bladder once removed and observed with a binocular loupe, the scale bar represents 1 mm. Black arrows indicate vascular lesions, and the dotted lines indicate the outline of the vascular lesions **C** Detail of vascular lesions according to the groups at 6 months post-irradiation (for each group and time *n* = 3). **D** Detail of vascular lesions according to the groups at 12 months post-irradiation (*n* = 3). **E** Analysis of micturition at 6 months (*n* = 8). A statistical test (ANOVA) was performed, *p* < 0.001 (***), *p* < 0.0001 (****)

To study the effect of MSC treatment on CRC, a group of 30 animals was not irradiated and not injected (unirradiated untreated group), a group of 30 rats was irradiated at 40 Gy and not injected (irradiated untreated group), a group of 30 rats was not irradiated and injected (unirradiated treated group), and a group of 30 rats was irradiated at 40 Gy and injected with three intravenous injections of 5 million of MSC (per rat) at intervals of two weeks, 4.5, 5 and 5.5 months post-irradiation (irradiated treated group). For each group, 10 animals were euthanized at 6, 8 and 12 months post-irradiation. The MSC injection protocol was designed to maximize the treatment effect according to previous studies. Cystoscopies at 6 and 12 months were performed to visualize vascular lesions (Fig. 1G). Hematuria was monitored once a month after irradiation (Fig. 2).

### Analysis of hematuria, vascular lesions and micturition

The rats were weighed and then placed in a metabolic cage once a month for 18 h and their urine was collected (as previously described [25]). The urine was analyzed using urine strips (URITOP 10 parameters, Exacto PRO^®^ from Biosynex). Analysis of micturitions was performed as previously described [25], a filter (Whatman filter paper 602H, 10312620 from Merck) is placed in the metabolic cages and the rats remain on these filters for an additional 4 h. The filters are then collected to count the number of spots is measured under UV lamp. If at any point in the study one of the rats has damaged the Whatman paper (making it impossible to analyze the number of voids) then it cannot be included for the specified time. Vascular lesions were analyzed by endoscopy at 6 and 12 months post-irradiation (Karl Storz cystoscope, 11583A).

### Isolation, characterization and culture of MSCs

GFP-MSCs were extracted from adipose tissue obtained from seven-week-old eGFP transgenic rats as described previously [26]. All Sprague–Dawley GFP + rats come from our institute. Briefly, subcutaneous inguinal adipose tissue was harvested from eGFP-SD rats, finely cut and enzymatically digested with 0.1% type I collagenase (reference: C0130, Sigma-Aldrich, St-Quentin-Fallavier, France) at 37 °C, and then filtered through a 40 µm filter. This process was repeated three times, and the collagenase was neutralized with 10% fetal bovine serum (FBS). Cells were washed with phosphate-buffered saline (PBS plus 10% SVF) and suspended in MEM-α containing 20% FBS, 1% penicillin/streptomycin and 1% L-glutamine. The cells were seeded at 1000 cells/cm^2^, and the medium was changed every 4 days. After 11–13 days, cells were trypsinized (trypsin-0.25% EDTA, Thermo Fisher Scientific), washed three times with PBS and suspended at 1 million cells/mL. The phenotype of the amplified Ad-MSCs was verified by flow cytometry. The presence of CD29 (clone Ha2/5; BD Biosciences), CD90 (clone OX-7; BD Biosciences, Le Pont de Claix, France) and CD73 (clone 5F/B9; BD Biosciences), and the absence of CD34 (clone ICO115; Santa Cruz Biotechnology, Inc., Dallas, USA) and CD45 (clone OX-1; BD Biosciences) were analyzed. The ability to form colony-forming unit fibroblasts (CFU-F) was also analyzed. CFU-Fs were stained with crystal violet and counted 11 days after initial seeding.

### Immunohistology

Bladders were collected and preserved in PFA (paraformaldehyde) and incubated for 12 h (fixation) before being processed for paraffin embedding. Sections of 5 μm were taken. These sections were subjected to hematoxylin and eosin (H&E) staining and immunohistochemistry. On H&E sections, lesions characterized as hyperplasia (abnormal thickening of the urothelium with hyperchromasia) were measured. For this purpose, a ratio of urothelium with hyperplasia to total urothelium length was performed.

Quantification of mast cells was performed by toluidine blue staining. Mast cells were counted and reported per unit area (in mm^2^), and then the group of unirradiated untreated animals is normalized to 1 and the other groups were compared to it, as previously described [25]. Animals with partial or total submucosal degradation were removed from the study in order not to influence the results.

Basal stem cells were labeled with cytokeratin 14 (ab119695 from abcam; 1:100 dilution, citrate buffer) and co-labeling with a proliferation marker (PCNA: ab29 from abcam; 1:100 dilution; citrate buffer) was performed. Antibody incubation was performed overnight. Secondary antibodies used were an Alexa Fluor 568 goat anti-rabbit antibody (for CK14) and an Alexa Fluor 488 goat anti-mouse antibody for PCNA. A ratio between the number of basal stem cells and the total number of cells was performed and then the group of unirradiated animals was normalized to 1 and compared to other groups. Animals with severely degraded urothelium were removed from the analysis to avoid introducing bias.

The superficial cells were labeled with uroplakin III (Abcam, ref: ab78196; dilution: 1/500, citrate buffer pH 6, 1×) and then revealed using the horseradish peroxidase (HRP) system (Zytomed ref: ZUC070-100) and a grading was performed.

Pictures are realized with the nanozoomer S60 of Hamamatsu and analyze with Histolab (version 12.1.1) and NDPview (version 2.9.29) software. For toluidine blue and HES images, the zoom is 200× with a resolution of 0.46 μm/pixel. For uroplakin III and immunofluorescence, the zoom is 400× with a resolution of 0.23 μm/pixel. An average of 5–10 focus points (depending on the sample size) was taken. All images of the same study were processed with the same parameters.

### Grading of uroplakin III

**Fig. 3:**
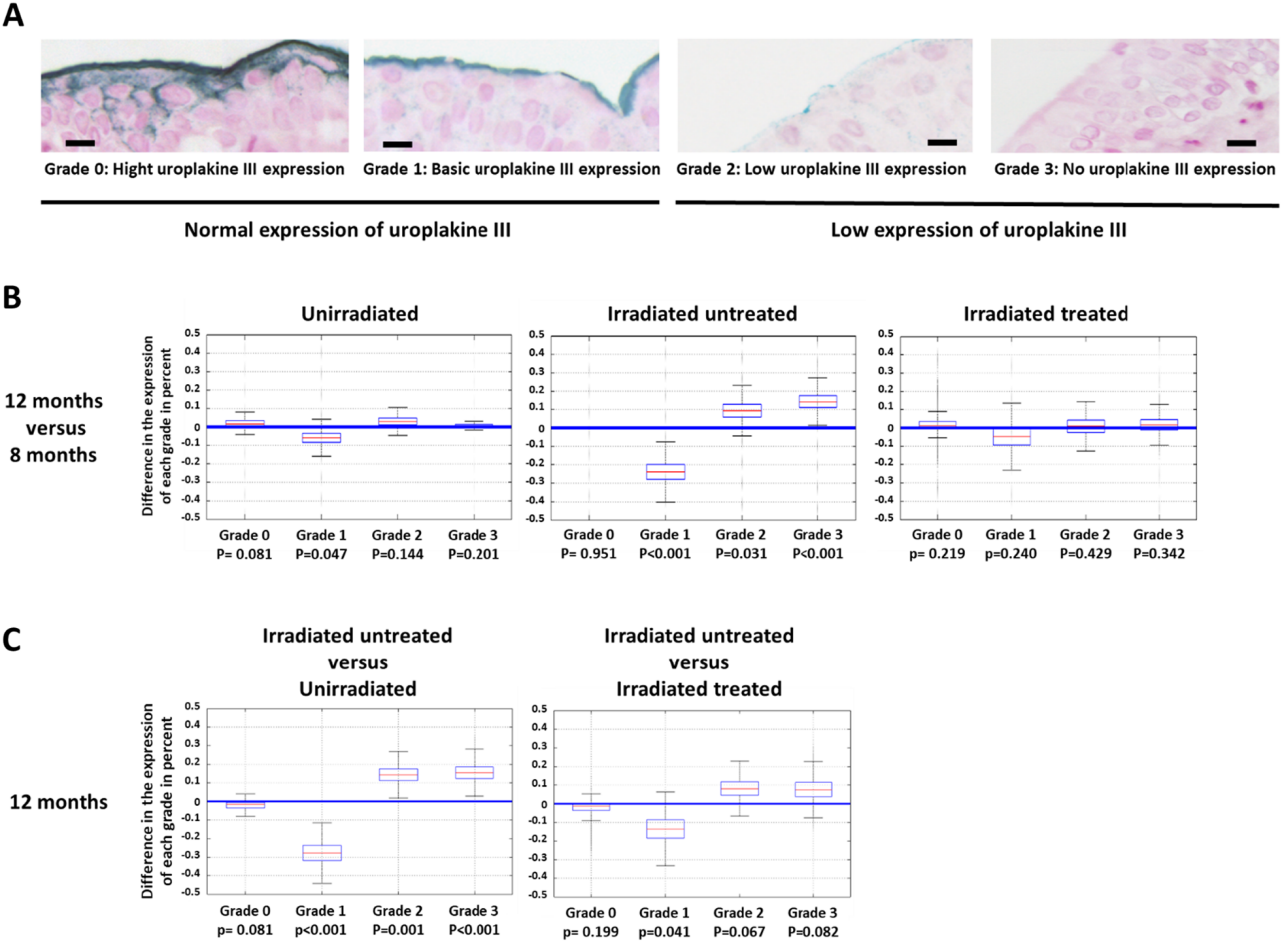
Evolution of uroplakin III grades as a function of time (at 8 and 12 months) and groups (non-irradiated, irradiated untreated and irradiated treated). Uroplakin grading was performed on the entire bladder of each rat. Each grade is expressed as a percentage with a total of 100% (combination of grades 0, 1, 2 and 3). **A** Representation of the different grades of uroplakin III expression. The black bar represents 10 μm. **B** Time-dependent variation of uroplakin III grade expression (for each group *n* = 10). **C** Variation in uroplakin III grade expression between groups (for each group *n* = 10). The boxplots represent the estimates of the grade probabilities with their associated uncertainties

The uroplakin III labeling was divided into four grades based on previous studies [27]. The grades were divided according to the level of intensity of the labeling. Grade 0 corresponds to strong expression of uroplakin III. The labeling is intense, thick and covers the entire surface of the superficial cells. Grade 1 corresponds to thin labeling and only on the apical part of the superficial cells. Grade 2 corresponds to a weak marking, the marking is discontinuous, and not very intense. Grade 3 is an absence of uroplakin III expression (see Fig. 3). Grades 0 and 1 correspond to a functional urothelium and Grades 2 and 3 to a urothelium with low uroplakin expression favoring a decrease in urothelial permeability. The entire urothelium was analyzed for each rat. The grading was performed on the whole urothelium, which gives a percentage of each grade for an individual.

### Statistical analysis

For the vascular lesions, statistical analyses were achieved using SigmaPlot v11 (Systat Software GmbH, Erkrath, Germany). Two-group comparisons were performed with *t* tests, while two-way ANOVAs followed by Bonferroni *t* tests were used for multiple group comparison. Results are expressed as mean ± standard error of the mean (SEM). A value of *p* ≤ 0.05 is considered to be statistically significant. In the figures, asterisks correspond to *p* < 0.0001 (****), *p* < 0.001 (***), *p* < 0.01 (**) and *p* < 0.05 (*).

## Results

### Irradiation induces an increase in hematuria from 40 Gy upwards as early as 100 days post-radiation

Hematuria is the main symptom used to measure the evolution of CRC. The criterion of the appearance of hematuria was used to evaluate the kinetics of the evolution of the chronic phase of CRC as a function of the radiation dose. Whole bladder irradiation in a dose range of 20–80 Gy (20, 30, 40, 50, 60, 70 and 80 Gy) was used to determine the optimal dose to induce hematuria.

In order to model the bivariate dynamics (in dose and post-irradiation time) of the evolution of CRC, a flexible nonlinear regression via spline functions was conducted (REFUND package of the R software). The result of this modeling is represented in Fig. 2A as a three-dimensional surface (and its 2D projection as a heat map). On the *x*-axis is the irradiation dose, on the *y*-axis the post-irradiation time, and the cumulative dose of erythrocytes per microliter (noted ery/μl) of urine in vertical dimension. In our model, at a dose of 20 Gy, hematuria appears late, beyond 200 days (6 months) after irradiation. At 40 Gy, hematuria appears as early as 100 days post-irradiation (100 ery/μl), indicated by the white arrows. This flexible bivariate modeling thus revealed a significant association between the irradiation dose and the extent of hematuria (*p* < 0.001). For doses of 60–80 Gy, hematuria also began at 100 days post-irradiation and increased up to 300 days post-irradiation (up to 200 ery/μl). Doses above 40 Gy are too deleterious (weight loss, rat grimace scale) to allow long-term follow-up of the rats. Thus, the results of the data modeling described above suggest quite clearly the 40 Gy dose for this study, as it induces early hematuria without impacting survival beyond 10 months.

### Reduction of vascular lesions after MSC treatment

Cystoscopy is used to describe lesions such as telangiectasias, ulcers, papillae and cancerous lesions in patients. It enables discrimination from other etiologies of hematuria, such as renal lesions, primarily. We used cystoscopy in our study model to objectify vascular lesions, to record the number and severity of vascular lesions and to detect any abnormalities (papilla, ulcer).

In Fig. 2B, images of cystoscopies (with lesions circled with a black dotted line) at 6 and 12 months are shown, and in Figs. 2C and D, the number of vascular lesions is shown as a function of the importance of the lesions and the treatment conditions. On the ordinate, the number of vascular lesions is given, and on the abscissa, the different groups as well as the total lesions. A statistical test (ANOVA) was carried out on all the data.

Figure 2C, at 6 months post-irradiation, an absence of vascular lesions was observed for unirradiated and untreated rats. For unirradiated and treated rats, 2 ± 1.1 lesions (Fig. 2C) were observed on average. For untreated irradiated rats, the animals had an average of 4 ± 4.7 lesions. For irradiated and treated rats, on average 5.7 ± 2.9 lesions were observed.

At 12 months post-irradiation, we can observe for unirradiated untreated rats an average of 0.7 ± 0.4 lesions (Figs. 2B, D). For unirradiated untreated rats, no vascular lesions are observed. For the irradiated untreated rats, the animals have an average of 4.3 ± 2.4 lesions. For irradiated and treated rats, an average of 1.3 ± 0.4 lesions is observed.

In conclusion, at 12 months post-irradiation, treatment with MSCs appears to reduce vascular lesions.

The data in Fig. 2E show that irradiation increases the number of micturition, but treatment significantly reduces micturition at 6 months. For unirradiated untreated rats, the number of micturition has an average of 3.5 ± 0.38 micturitions. For the irradiated untreated rats, the animals have an average of 9.75 ± 1.22 micturitions. For irradiated and treated rats, an average of 3.12 ± 0.51 micturitions is observed. Comparing irradiated untreated rats with unirradiated rats at 6 months an increase in micturitions of 2.79 fold is observed (*p* value < 0.001). Comparing irradiated treated rats with irradiated untreated rats at 6 months, a decrease in micturitions of 3.1 fold is observed (*p* value < 0.0001). Results are in favor that treatment might restore functionality of bladder after irradiation.

### MSCs have a protective effect on the impermeability of the urothelium

MSC treatment restores the structure and functionality of the epithelia after irradiation at the intestinal level [24]. In our study model, we evaluated whether MSC treatment protects the urothelium from irradiation. The function of the uroplakin complex is to ensure the impermeability of the urothelium. A decrease in uroplakin expression is directly related to a loss of urothelial impermeability [28]. Uroplakin III labeling was performed by visible immunohistochemistry. A grading system for uroplakin III expression was set up and measured on the whole urothelium of each animal, as described in the Methods and Materials section. Grade 0 indicates a high level of uroplakin, while Grade 3 indicates no uroplakin (Fig. 3A). The lower grades therefore indicate a urothelium that has retained a normal level of uroplakin III while the higher grades indicate a degraded urothelium. Thus, for each animal the percentage of urothelium in Grades 0–3 was quantified.

A rigorous statistical analysis of the differences in grade percentages cannot therefore be based on standard analysis tools (ANOVA, *t* tests, etc.) due to the very strong constraints of the input data: the percentages of Grades 0–3 for each animal are not only data between 0 and 1 but have the additional condition of having a sum equal to 1. In order to take this data format into account, a compositional analysis [29] was conducted, which first consisted in transforming the percentage vectors into unconstrained data in order to deploy the classical analysis tools. Due to the presence of zero percentages, a spherical transformation [30] was used.

Figure 3B is a kinetic study of the evolution of the grades comparing 12 months to 8 months for each condition (e.g., 8 months non-irradiated rats compared 12 months non-irradiated rats). Figure 3C shows the difference in uroplakin III expression grades at 12 months for irradiated untreated rats compared to unirradiated rats (Fig. 3C left) and for irradiated untreated rats compared to irradiated treated rats (Fig. 3C right).

When comparing the unirradiated rats at 12 months compared 8 months, there is no change in grades over the course of time (Fig. 3B, unirradiated). When comparing the irradiated untreated rats at 12 months compared 8 months a decrease in Grade 1 by 25% (*p* value < 0.001), an increase in Grade 2 by 10% (*p* value = 0.031) and Grade 3 by 15% (*p* value = 0.001) is observed (Fig. 3B, irradiated untreated). In contrast, the analysis revealed no significant changes in uroplakin III grades in irradiated and treated rats over time (Fig. 3B, irradiated treated). In summary, treatment with MSCs inhibited the effects of irradiation over the course of time (no increase in grades).

Comparing irradiated untreated rats with unirradiated rats at 12 months a decrease in Grade 1 of 27.5% (*p* value < 0.001), an increase in Grade 2 of 14.25% (*p* value < 0.001) and Grade 3 of 15.5% (*p* value = 0.001) is observed (Fig. 3C irradiated untreated compared unirradiated). Irradiation therefore induces the loss of uroplakin III expression after irradiation (Fig. 3C irradiated untreated compared irradiated treated).

Comparing irradiated untreated rats with irradiated treated rats at 12 months shows, for the treated irradiated rats compared to the irradiated rats, a significant decrease in Grade 1 of 13.5% (*p* value = 0.041), and an increase close to significance in Grades 2 and 3 is observed for the untreated rats (8%, *p* value = 0.067 and 7.5% *p* value = 0.082 respectively). Treatment with MSCs thus appears to limit the loss of uroplakin III expression after irradiation.

In conclusion, irradiation increases the loss of uroplakin III expression (Grade 2 and 3). In contrast, MSC treatment limits the loss of uroplakin III expression (Grades 2 and 3) induced by irradiation. MSCs therefore appear to have a protective effect on uroplakin III expression by maintaining its expression over time in treated irradiated rats compared to untreated irradiated rats. These results suggest a protective effect of MSCs on the maintenance of uroplakin III expression and thus a maintenance of urothelial impermeability.

### MSCs allow a reduction in irradiation-induced hyperplasia

**Fig. 4:**
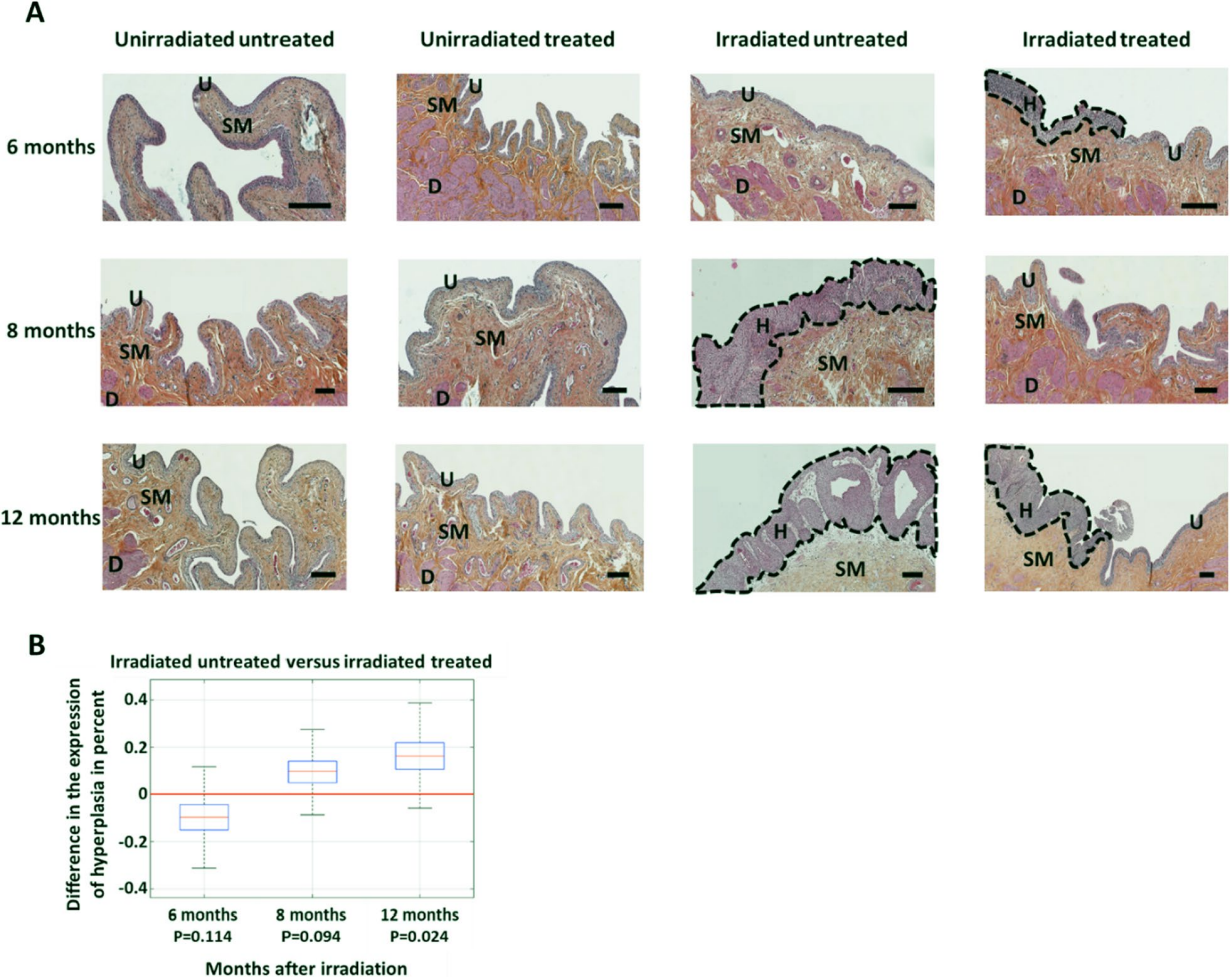
Study of hyperplasia as a function of time and groups. **A** Representative photograph of hyperplasia as a function of time and groups. The black bar represents 100 μm. U = urothelium, SM = submucosa, D = detrusor, H = hyperplasia (hyperplasias are outlined by the black dotted line). **B** Difference in the probability of hyperplasia between the group of untreated and treated irradiated rats after irradiation (for each group *n* = 10). The boxplots represent the estimates of the grade percentages with their uncertainties

Hyperplasia is deleterious in the long term as it permanently reduces impermeability and can induce lesions such as metaplasia or precancerous lesions [31]. The aim of the hyperplasia analysis is to assess whether MSC treatment protects the functional integrity of the urothelium as well as preventing regeneration abnormalities. The length of the urothelium with hyperplasia was measured and related to the total size of the urothelium which represents a percentage of the length of the urothelium with hyperplasia, as previously defined [32]. Figure 4A shows representative pictures of the hyperplasia (H) visible on the HES sections. For unirradiated rats (treated or untreated, Fig. 4A, to the left), no hyperplasia was noted in the urothelium (U). In contrast, rats irradiated from 8 months of age showed strong hyperplasia (H) which increased at 12 months (Fig. 4A, irradiated untreated, on the right). When the rats are treated with MSCs, the hyperplasia is more limited and restricted to limited areas (Fig. 4A, irradiated treated, on the right).

As with the uroplakin III grading analyses, compositional analysis based on spherical transformation allows the effect of MSC treatment on urothelial hyperplasia to be assessed (Fig. 4B) as a function of time after irradiation. It appears that treatment with MSCs limits hyperplasia over time compared to the untreated irradiated group. Only the 12-month data are significant and show that the untreated irradiated rats have 16% more urothelium with hyperplasia compared to the MSC-treated irradiated rats (*p* value = 0.024).

**Fig. 5:**
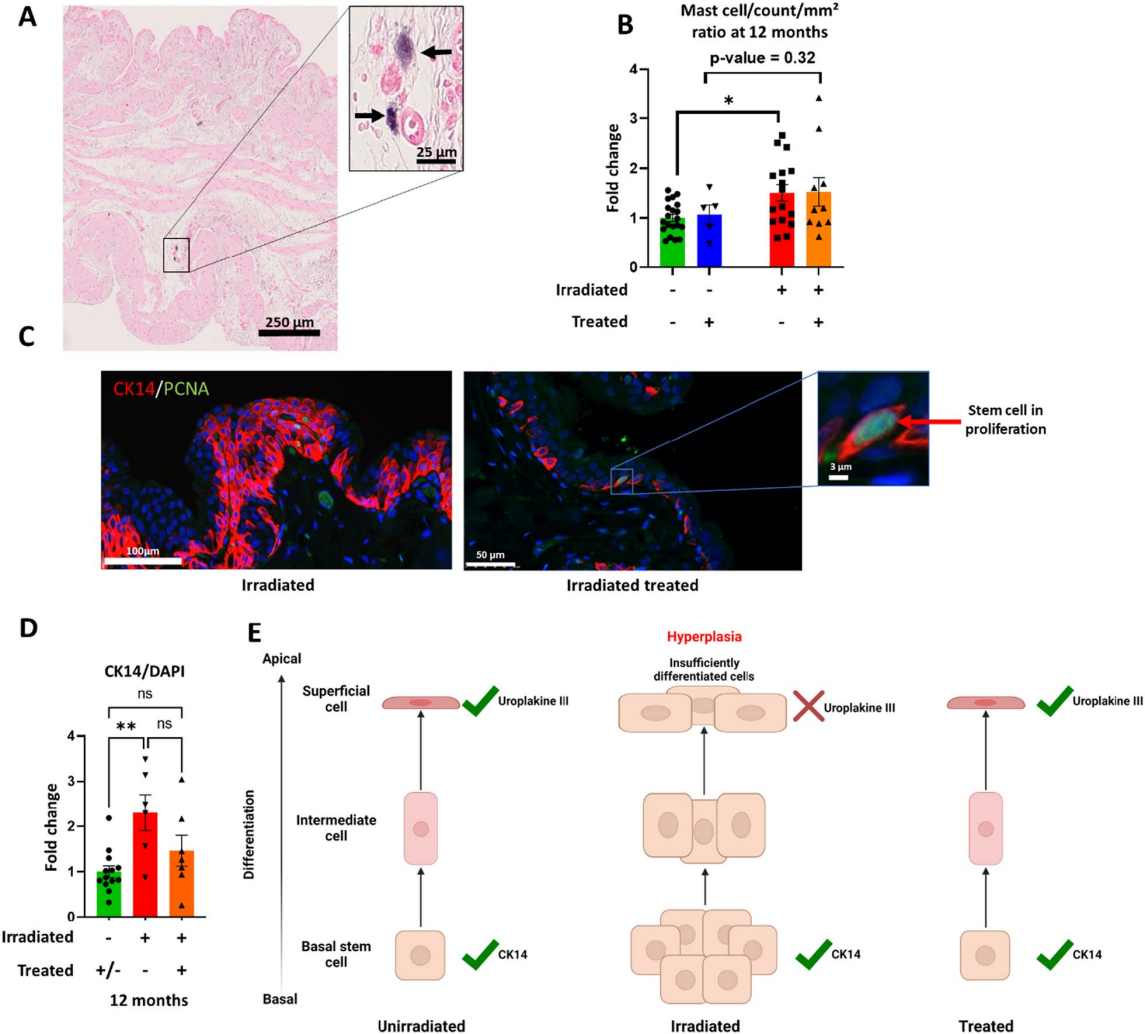
Study of inflammation and urothelium regeneration. **A** Bladder section stained with toluidine blue 12 months. Dark arrows indicate mast cells. **B** Analysis of the number of mast cells per unit area for each group and normalized to unirradiated untreated (unirradiated untreated group *n* = 20; unirradiated treated group *n* = 5; irradiated untreated *n* = 16; irradiated treated *n* = 10). **C** Immunohistochemistry of basal stem cells (in red) and their proliferation (PCNA in green) according to irradiated and treated groups. The image of the irradiated group represents an area with hyperplasia. Red arrow indicates a basal stem cell in proliferation. **D** Ratio of basal stem cell number (red) to total cell number (DAPI) according to group at 12 months after irradiation. The group of unirradiated and untreated rats was normalized to 1, and then, the other groups were compared to it (unirradiated group *n* = 13; irradiated untreated *n* = 6; irradiated treated *n* = 7) **E** Diagram explaining the hyperplasia phenomena and their consequences on the presence of CK14 and uroplakin III. A statistical test (ANOVA) was performed, *p* < 0.01 (**)

Hyperplasia may be mediated by inflammation. Mast cells are in part responsible for the inflammation in CRC. To investigate whether mast cells could be responsible for the hyperplasia, and whether MSCs could down-regulate this effect, we quantified the number of mast cells (Fig. 5A, B). For irradiated untreated rats, an increase in the number of mast cells (fold change = 1.5 ± 0.56; *p* value < 0.05) compared with untreated unirradiated rats is observed. For treated irradiated rats, the number of mast cells is not significantly different from the group of irradiated rats. In conclusion, after irradiation there is an increase in mast cells in bladder of untreated irradiated rats. Nevertheless, no observable effect of MSC treatment was observed. This is an indication that inflammation might be related to hyperplasia nevertheless MSC treatment, does not appear to influence inflammation with respect to the number of mast cells.

We retained to go further on the understanding of the mechanistic to support the observations of the integrity of the urothelium and hyperplasia. As shown in Fig. 5C, in the irradiated group of rats, hyperplasia is observed, due to a strong proliferation of basal stem cells without differentiation into superficial cells compared to the treated group. The superficial cells ensure the impermeability of the urothelium through the expression of uroplakin 3. We quantified the number of basal stem cells (labeled with cytokeratin 14) as shown in Fig. 5C. The results in Fig. 5D confirm that irradiation increases the number of basal stem cell, and that treatment is in favor of decreased number of basal stem cells. For irradiated untreated rats, an increase in the number of basal stem cells (fold change = 2.31 ± 0.4; *p* value < 0.1) compared with untreated unirradiated rats is observed. For treated irradiated rats, the number of basal stem cells is not significantly different from the control group (fold change = 1.46 ± 0.34).

In conclusion, MSCs induce a slight transient hyperplasia at 6 months post-irradiation (not significant) which may be associated with early regeneration of the urothelium. At longer times (8 and 12 months), the group of irradiated rats showed a strong hyperplasia which the MSC treatment seemed to have regulated. This process does not seem to be induced by down regulation of inflammation by MSCs but rather by controlling the regulation of urothelium regeneration. These results therefore suggest that MSC treatment reduces the intensity of hyperplasia during the course of CRC.

## Discussion

The aim of this study was to find out whether MSCs are a therapeutic alternative for limiting the progression of CRC. First, it was necessary to set up a rat model of radiation cystitis. The treatment was then carried out under optimal conditions according to previous studies [24, 33–37]) to study whether the systemic injection of MSCs from adipose tissue can limit the progression of radiation cystitis in the chronic phase. As hematuria is the hallmark of CRC progression, this parameter was chosen to set up the model of CRC. Then, in order to evaluate the effect of MSC treatment, the two compartments responsible for the chronic evolution of radiation cystitis were targeted for the continuation of this study, namely the vascular compartment and the urothelium [38].

For preclinical models of radiation cystitis, there are several irradiation configurations. In the majority of previous studies, exposure of the abdominal area resulted in exposure to irradiation of other organs. These irradiated organs could interact with the bladder [39]. Recent studies using the SARRP platform have allowed irradiation of the bladder only [25, 40, 41]. Based on these recent studies, in order to induce chronic radiation cystitis, a single-dose irradiation of the entire bladder was performed using SARRP, with the female Sprague–Dawley rat as an animal model. The use of rats for our study instead of mice is justified by the use of rat MSCs, whose characteristics are closer to human MSCs than to mouse MSCs [42, 43]. We chose female rats in order not to have the prostate in the irradiation field.

To determine the optimal irradiation dose to induce CRC in our model, a dose range of 20–80 Gy was performed. The kinetics of the evolution of hematuria allowed us to determine that its intensity is proportional to the dose received at the bladder, with a threshold at the dose of 40 Gy. The 40 Gy dose has the advantage of inducing vascular lesions without causing deleterious lesions for the animal, unlike higher doses. This dose is consistent with the literature where it is described to induce CRC [25, 40, 41, 44, 45], justifying the 40 Gy dose for the MSC study.

The aim is to evaluate whether MSC treatment limits the progression of radiation cystitis in its chronic phase. Our previous studies have shown that intravenous MSC treatment is optimal with three intravenous injections of 5 million MSCs from adipose tissue [33] at two-week intervals. This protocol significantly reduced colorectal lesions after irradiation [24, 33–37]. It reduces fibronecrosis and edema if the injection is carried out during the initial fibrous phase. [34]. Based on our previous studies, we studied the role of three intravenous injections of MSCs during the phase preceding the chronic phase of cystitis in order to potentiate the effect of MSCs.

Cystoscopy showed that MSC treatment is in favor of reduction of vascular damage 12 months after irradiation to the same level as the non-irradiated control. MSC treatment thus seems to induce a reduction of vascular lesions in the long term. These results are in agreement with preclinical studies on other types of cystitis, which show that MSC treatment induces neovascularization which may participate in the reduction of hematuria [16, 18]. Clinical trials in HC patients confirm the role of MSCs on vasculature, as they decrease or stop long-term hematuria in patients, thus highlighting the effectiveness of MSCs on the vascular compartment [2–4].

The loss of impermeability of the urothelium, due to defective repair, no longer allows protection of the underlying tissues. This mechanism could be at the origin of the chronic phase of radiation cystitis [14]. At the intestinal level, intravenous injection of MSCs induces proliferation of endogenous progenitor cells of the intestinal crypts allowing colonic epithelial regeneration after irradiation [24, 37, 46–48]. Based on the literature results showing that MSC treatment accelerates epithelial regeneration after irradiation, we investigated whether MSC treatment would enable urothelial regeneration after irradiation.

An increase in the number of hyperplasias was observed in irradiated rats over time, confirming previous studies [19, 25, 45]. Treatment with MSCs induces a slight transient increase in hyperplasia which may be due to regeneration at 6 months after irradiation in the initial phase of CRC [24]. These results suggest that MSCs induce an action as early as 6 months post-irradiation. In the long term, the effect of MSC treatment is a decrease in hyperplasia. Our results are in agreement with previous studies which show that during IC, MSC treatment induces a complete regeneration of the urothelium with a decrease in edema, denudation and regeneration of the lamina propria and the basement membrane [1, 22]. By protecting the urothelium, MSCs could therefore play a major role in limiting the progression of CRC.

The superficial layer of the urothelium is composed of superficial cells which are also called umbrella cells. These have a membrane composed of uroplakins which ensure the impermeability of the bladder. Preclinical studies show, during CRC, lesions of the urothelium in relation to a decrease in the expression of uroplakin III. The consequence is a loss of bladder tightness and impermeability [19, 20, 25]. In our model, treatment with MSCs maintains the level of uroplakin III expression over time from 8 months after irradiation. These results are in agreement with studies on acute interstitial cystitis, where MSC treatment increased uroplakin III expression [22, 23].

## Conclusion

In conclusion, our results support the protective action of MSCs in reducing hyperplasia and restoring homeostasis and impermeability of the urothelium. Figure 5E illustrates the proposed mechanism. In the non-irradiated group, the slow renewal of the urothelium is ensured by the division of basal stem cells and their successive differentiation into intermediate cells then into superficial cells which ensure impermeability thanks to the expression of uroplakin 3. In the irradiated group, areas of hyperplasia were observed. They are due to a strong and deregulated proliferation of basal stem cells without differentiation. The treatment with MSC would allow to rebalance the tissue homeostasis by promoting differentiation of superficial cells.

## Data Availability

The data that support the findings of this study are available from the corresponding author upon reasonable request.

## Abbreviations

CRC: Chronic radiation cystitis
MSC: Mesenchymal stem cell
Gy: Gray
PFA: Paraformaldehyde
H&E: Hematoxylin eosin
SARRP: Small animal radiation research platform
FBS: Fetal bovine serum
PBS: Phosphate-buffered saline
